# Multi-Floor Indoor Pedestrian Dead Reckoning with a Backtracking Particle Filter and Viterbi-Based Floor Number Detection

**DOI:** 10.3390/s21134565

**Published:** 2021-07-03

**Authors:** Cedric De Cock, Wout Joseph, Luc Martens, Jens Trogh, David Plets

**Affiliations:** Department of Information Technology, IMEC-WAVES/Ghent University, Technologiepark-Zwijnaarde 126, 9052 Gent, Belgium; wout.joseph@ugent.be (W.J.); luc1.martens@ugent.be (L.M.); jens.trogh@ugent.be (J.T.); david.plets@ugent.be (D.P.)

**Keywords:** pedestrian dead reckoning, indoor localisation, smartphone, inertial measurement unit, particle filter, DBSCAN, barometer, WiFi, floor transitioning, Viterbi

## Abstract

We present a smartphone-based indoor localisation system, able to track pedestrians over multiple floors. The system uses Pedestrian Dead Reckoning (PDR), which exploits data from the smartphone’s inertial measurement unit to estimate the trajectory. The PDR output is matched to a scaled floor plan and fused with model-based WiFi received signal strength fingerprinting by a Backtracking Particle Filter (BPF). We proposed a new Viterbi-based floor detection algorithm, which fuses data from the smartphone’s accelerometer, barometer and WiFi RSS measurements to detect stairs and elevator usage and to estimate the correct floor number. We also proposed a clustering algorithm on top of the BPF to solve multimodality, a known problem with particle filters. The proposed system relies on only a few pre-existing access points, whereas most systems assume or require the presence of a dedicated localisation infrastructure. In most public buildings and offices, access points are often available at smaller densities than used for localisation. Our system was extensively tested in a real office environment with seven 41 m × 27 m floors, each of which had two WiFi access points. Our system was evaluated in real-time and batch mode, since the system was able to correct past states. The clustering algorithm reduced the median position error by 17% in real-time and 13% in batch mode, while the floor detection algorithm achieved a 99.1% and 99.7% floor number accuracy in real-time and batch mode, respectively.

## 1. Introduction

Indoor localisation has many applications, such as tracking objects or pedestrian navigation. Satellite navigation systems, e.g., GPS, are not usable indoors. Dedicated localisation systems for indoor environments have been developed in recent years [1,2]. These systems make use of wireless technologies, such as Ultra-Wideband (UWB) [3,4,5], visible light communication [6,7], WiFi [8,9] or Bluetooth [10]. A human or object is equipped with a tag that is localized relative to a fixed infrastructure of anchor nodes. A common drawback of these systems is dependency on the anchor nodes [2]. These are expensive, and their setup requires manual work. The accuracy of these systems is affected by (changes of) the environment, as well as the placement and quantity of anchor nodes.

An alternative solution for indoor localisation is Inertial Navigation Systems (INSs), using Inertial Measurement Units (IMUs). These devices consist of three-axis accelerometers, gyroscopes and, optionally, magnetometers. The smartphone has been widely adopted by the public during the last decade, and most smartphones are equipped with an IMU. While more accurate systems use a dedicated IMU strapped to the foot or leg [11], smartphone-based systems have some obvious advantages: they can be deployed without dedicated hardware and offer good user comfort because people already carry their phone with them. A smartphone-based INS starts with Pedestrian Dead Reckoning (PDR). In this method, individual steps are detected from acceleration or gyroscope data. For each step, the step length and step heading are estimated by fusing the accelerometer, gyroscope and/or magnetometer data. The trajectory is then estimated by dead reckoning [12,13]. The major disadvantage of INS/PDR is that errors are cumulative: the localisation error increases with time. A challenging aspect is heading estimation. Most approaches rely on using only the gyroscope (delivering good heading accuracy in a short time interval, but prone to drift over time due to integration errors) [14,15,16] or the fusion of the gyroscope with the magnetometer (delivering absolute headings, but prone to large errors due to the presence of iron materials in indoor environments) [17,18,19].

The output of a PDR algorithm is often matched to a scaled floor plan by a Particle Filter (PF) [20,21,22,23,24]. Wall locations are used as physical constraints, eliminating drift caused by both heading and step length estimation errors. In fact, the PF with map matching can track the pedestrian even if the initial position and/or heading are unknown [20,23,24]. Common problems with the PDR-PF approach are sample impoverishment [25] and multimodality [19,23]. The former happens when the filter relies too much on the output of the PDR algorithm. The particles can become stuck in one place due to errors in the PDR algorithm. The latter happens when the filter is allowed to diverge too much from the PDR output. The particles can then spread out into different modes. Furthermore, multimodality can also be caused by symmetry in the walkable environment [26]. These problems can be solved by fusing the PDR-PF system with the mentioned localisation techniques into a hybrid localisation system. The tradeoff is that these hybrid systems are dependent on dedicated infrastructure, which is not always available.

Another challenge in an indoor pedestrian localisation system is detecting floor transitions and determining the correct floor number (i.e., floor detection), thus allowing a pedestrian to be tracked across multiple floors. This can be achieved by detecting the floor number directly using (WiFi) fingerprinting [25,27] or by detecting floor transition events using data from onboard sensors [24,28,29,30,31].

We designed and implemented a complete smartphone-based indoor pedestrian localisation system, which is independent of any dedicated localisation system and does not require knowledge of the pedestrian’s initial position, heading and floor number. The system consists of a PDR algorithm [17] and the Backtracking Particle Filter (BPF) [32]. Clustering the particles with Density-Based Spatial Clustering for Applications with Noise (DBSCAN) [33] was used on top of the BPF, increasing the accuracy when the particle distribution is multimodal. We proposed a new accurate floor detection algorithm based on the Viterbi algorithm, which actively detects both stairs and elevator usage (and vertical direction) without the need for additional/dedicated hardware (except the smartphone being carried). Received Signal Strength (RSS) Model-based Fingerprinting (MBF) using only pre-existing WiFi Access Points (APs) provides rough floor number detection and was fused with the PDR-BPF localisation system. The BPF and Viterbi-based floor detection algorithm keep track of past positions and floor numbers, respectively, and can correct them using new information. Therefore, this system is best suited for applications where a delay of the output is acceptable. The only requirement for this system is that a detailed floor plan and some WiFi APs be present, which is the case for most public buildings and office environments.

The contribution of this paper was an infrastructure-independent smartphone-based multifloor indoor pedestrian localisation system with:

- The combination of clustering, MBF of WiFi RSS, detection of both stairs and elevator usage and backtracking to reduce the multimodality problem with particle filters in the indoor PDR context and the step length and heading drift errors from the PDR algorithm;
- Floor number detection via WiFi RSS MBF and a floor transition detection algorithm, detecting both stairs and elevator usage by fusing accelerometer and barometer data;
- Integration into a complete infrastructure-independent localisation system, able to track pedestrians across multiple floors.

The remainder of the paper consists of related works (Section 2), then the methodology and implemented algorithms are explained (Section 3) and evaluated (Section 4). The work concludes with a discussion in Section 5.

## 2. Related Work

### 2.1. Pedestrian Dead Reckoning

While some works presented full PDR systems, others focused on the subproblems of PDR: determining device orientation, step detection, step length estimation and pedestrian heading estimation. The orientation is used to transform sensor measurements from the local to the global coordinate frame. This is necessary to estimate the pedestrian’s heading. Generally, the accelerometer is used to estimate the gravity vector, and thus the tilt. The magnetometer data can then be tilt compensated and provide the device heading. However, the attitude from accelerometer data is sensitive to other forces (e.g., from walking), and the magnetometer is sensitive to magnetic objects [34]. Attitude and Heading Reference System (AHRS) algorithms fuse gyroscope data with accelerometer and optionally magnetometer data for robust device orientation. Two known AHRS algorithms are the Madwick and Mahoney filters [35]. Kalman filters are also implemented as AHRS, of which the unscented KF was shown to have the best performance in [36]. The pedestrian heading depends on the carrying mode. Our system used the device heading directly as in [17,37], because the phone is always held in the hand without rotating it relative to the user’s body. Multimode PDR systems allow multiple carrying modes (e.g., in the pocket), of which many are based on Principal Component Analysis (PCA) of accelerometer data [28,38]. Since the device and pedestrian heading were identical in our system, they are simply called heading in the remainder of the text. Acceleration peak detection is the most popular step detection method [17,19,28]. Other methods are based on device attitude [37] or relative amplitudes in the frequency domain [39]. The step length can be modelled according to the peak-to-peak [40] or variance and peak frequency [19] of acceleration and pitch amplitude [37]. Several step detection and step length algorithms were compared in [39,41], respectively.

### 2.2. Map Matching with Particle Filters

In [20], it was demonstrated that the IMU in a typical smartphone is less accurate than a dedicated IMU (Xsens), but map matching improves their localization accuracy to the same level. Contrary to PDR algorithms, the differences in other PF implementations are more subtle. Reference [21] assumed the user is often walking in a straight line. When a set of particles is in a corridor and the heading change is small, the filter will guide the particles along the direction of the corridor. If the heading change is large and a door is nearby, the particles are guided towards the door. Detailed floor plans of public buildings are not always available. Reference [24] used the simplified Open Street Maps (OSM) floor plans and enhanced them, assuming interior properties (e.g., minimum corridor width) conformed to established standards. A mesh-based transition model was proposed in [23], which allowed propagation towards possible locations only by calculating all wall intersections once in an offline phase. This is similar to using graphs [42], but a mesh is also more memory efficient. Reference [22] used a Gaussian curve to weigh particles in addition to removing impossible particles. What makes map matching especially powerful is that it can be used when the initial position is unknown. The initial particles are then uniformly distributed over the floor plan [23,24]. When the initial heading is unknown, the particles are initialized with a random heading [20,23] or the magnetometer is used to estimate the initial heading [24].

As mentioned, a known problem with particle filters is the tradeoff between particle diversity and focus [43], determined by the amount of artificial noise added by the propagation model. Too much focus means the PF relies too much on the PDR output, causing all the particles to become stuck in the wrong room due to PDR errors and hard wall constraints. Large diversity means the PF can diverge heavily from the PDR output (and/or ignore physical constraints), causing multimodal state distributions. This multimodality problem is often mentioned [19,22,43], but mostly left unsolved. Reference [23] proposed an approximation of a Gaussian Kernel Density Estimator (KDE) to select the most probable mode of the multimodal particle distribution instead of averaging all particles. However, no global improvement was achieved. Smoothing with a forward–backward smoother [25] and Backtracking Particle Filter (BPF) [32] was proposed, which can correct past multimodality errors if the filter converges to one mode again in a later stage.

### 2.3. Hybrid Localisation

In hybrid localisation, two or more localisation techniques are fused to provide better accuracy than each separate technique. PDR is often fused with WiFi/Bluetooth localisation. Trilateration using RSS [44] or channel state information [45] has been proposed, but RSS fingerprinting is the most popular method [46,47,48]. Fingerprint databases or radio maps are constructed in an offline phase, where each position corresponds to a vector of RSS values of APs in range. The RSS vectors were acquired empirically in [44,48], while [46] used a path loss model (i.e., MBF). In the online phase, a KDE was used in [48] to estimate the location and error covariance matrix given the radio map and a new RSS vector. This was then fused with PDR in an unscented KF. In [44], fingerprints were matched with the Euclidean distance, and the chosen position was fused with trilateration using a KF. The resulting position was then fused with PDR using a second KF. The work in [48] used K Nearest Neighbours (KNNs) instead, and the resulting position was fused with PDR in the same PF used for map matching. After the PF has removed impossible particles, the remaining ones are weighted based on their distance to the estimated position from fingerprinting. Similarly, Reference [46] weighed each particle by matching the database RSS vector at the particle’s location to the new RSS vector. The rough, but absolute positioning provided by WiFi improved the accuracy, especially during the initial stages when particles were still spread out in different modes. Furthermore, sample impoverishment in PDR-PF systems can be solved in a hybrid localisation system. A KF as a second filter was proposed in [25], using only PDR and WiFi RSS measurements. When the deviation of the particle closest to the KF’s state estimate passes a threshold, the PF is reinitialized by sampling from the KF’s state distribution. Similarly, an Interacting Multiple Model Particle Filter (IMMPF) was proposed in [43], where a secondary PF uses only the WiFi RSS as the input. The main PF samples from the secondary PF when the Kullback–Leibler divergence between the two filter passes a threshold. In parallel, the secondary PF samples from the main PF when outliers in the RSS measurements are detected. Lastly, an infrastructure-independent hybrid localisation system was proposed in [49], which exploited the smartphone’s IMU and camera for fusion of PDR and camera-based Simultaneous Mapping and Localisation (SLAM).

### 2.4. Floor (Transition) Detection

In [27], the floor was chosen that had the database RSS vector with the highest similarity to the measured RSS vector. However, detection accuracy depends on the environment and the available APs. Floor transition detection using machine learning and features extracted from multiple sensors (accelerometer, gyroscope and/or barometer) was successfully implemented in [19,24,28,50]. References [28,29] obtained over 90% accuracy in distinguishing between going upstairs and downstairs, but elevators were not detected. References [24,50] detected both stairs and elevator usage, but it performed worse at distinguishing the direction of stairs usage. Floor (transition) detection can also be achieved by detecting height changes. The cumulative height change during a transition can then be used to estimate the amount of changed floors. Height change was accurately estimated in [29,51] using only the IMU sensors, but this was achieved using dedicated strapdown IMUs, which provide higher accuracy than unconstrained smartphones [20]. The barometer was used successfully as an alternative to detect floor transitions by converting the measured pressure to height [52]. Reference [31] used the height calculated from the first pressure measurement as a reference to estimate the height difference during the rest of the trajectory. However, the estimated height can drift by several meters within an hour due to atmospheric pressure drift caused by the weather [30]. In [53], the moving average and linearity of the pressure data were used to detect floor transitions. However, the pattern being recognized only applied to a specific type of staircase, and also, pedestrian detection using surveillance camera’s was used for higher accuracy. Accurate height estimation and floor (transition) detection was achieved in [30,54] with the barometer and a Kalman filter, but a reference barometer at a known floor compensated for atmospheric pressure changes over time. Without a reference device, for systems based on floor transition detection and/or relative height estimation, the initial floor number must be known, and it is difficult to recover from false or missed floor transitions. This was solved by adding absolute floor detection with WiFi (or Bluetooth) fingerprinting [23,25,55,56]. Reference [55] used WiFi RSS and barometer measurements in the update phase of a Kalman filter to estimate the height. References [56,57] used WiFi RSS for floor detection and a barometer to detect stairs. Reference [56] used a probabilistic model to detect if a recent pressure change was caused by a floor change. Therefore, a floor number change was only detected when the change was (almost) finished. Reference [57] used a moving average to detect floor transitions, but the floor number was detected with a pressure look-up table, which needed frequent recalibration using WiFi fingerprinting. Reference [23] used WiFi and Bluetooth fingerprinting and detected floor transitions with the barometer and gyroscope. However, their method depended on the placement of many APs (e.g., 42 beacons). In [25], fingerprinting was only used for 2D localisation. The barometer was used to detect height changes, while the PF was allowed to propagate particles on all floors. The floor number of the floor with the most particles was chosen.

## 3. Method

**Figure 1:**
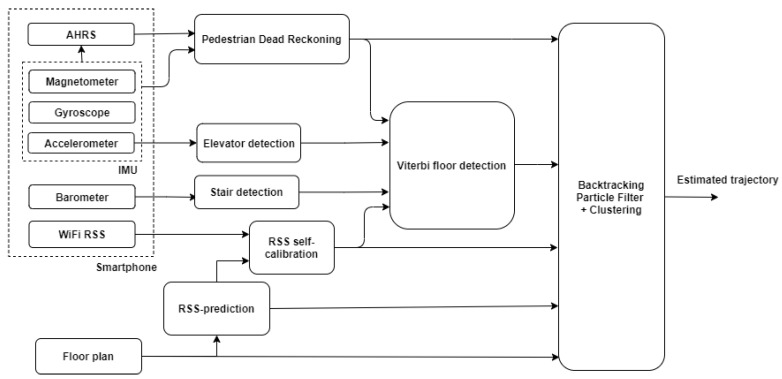
High-level flowgraph of the application.

Figure 1 illustrates a high-level overview of the algorithm explained in this section. Each block references the corresponding subsection. The following steps were realized: PDR (Section 3.1), WiFi RSS-MBF (Section 3.2), a BPF (Section 3.4) fusing PDR, WiFi RSS measurements and floor plan information and, finally, a Viterbi-based floor number detection algorithm (Section 3.3), including stairs and elevator detection.

### 3.1. Pedestrian Dead Reckoning

As illustrated in Figure 1, the data used for this part came from the smartphone’s IMU, which consisted of 3-axis accelerometers, gyroscopes and magnetometers. The PDR algorithm was reproduced from two papers and therefore only briefly explained. For more detail, see [17,41].

#### 3.1.1. Calibration and Preprocessing

The gyroscope was calibrated by placing the device on a stable surface for a few seconds and subtracting the average value of each axis from the consecutive measurements [58]. This removed the gyroscope bias. The magnetometer was calibrated by compensating for the influences of hard and soft magnetic objects, which cause additive and multiplicative errors, respectively. After rotating the device around two perpendicular axes, least squares ellipsoid-fitting was used as described in [59]. The smartphone can be tilted while holding it, and the tilt will inevitably fluctuate while the user is walking. This problem was solved by rotating the sensor data. The Android OS has a software AHRS sensor [60], which can provide the orientation as pitch, roll and yaw angles. Pitch and roll represent the tilt and were used to construct the rotation matrix. After rotating the accelerometer data, the Z-axis represents vertical acceleration and X- and Y-axes represent horizontal acceleration. The same applies for the magnetometer, for which the horizontal components were used to calculate the heading. The gyroscope heading was calculated by scalar projection of the gyroscope data onto the estimated gravity vector. This resulted in the horizontal angular rate, which was then integrated. For more details, see [17].

#### 3.1.2. Step (Length) Detection

When the pedestrian is walking, the vertical acceleration pattern resembles a sinusoidal wave. First, the gravity or DC component, as well as high-frequency noise are removed. Each step event is then detected by finding peaks in the data, which are caused by the impact of the foot on the ground. More details can be found in [17]. The step length $l_{n}$ of the n-th step was based on the model proposed in [41] (Equation (1)), which improved the known Weinberg model [40]. $a_{max,n}$ is the detected peak value, and $a_{min,n}$ is local minimum that precedes the peak. In the original model, the coefficient $K_{n}$ was constant. In [41], however, $K_{n}$ was estimated during each step, based on a quadratic function of the estimated velocity. The velocity was estimated by integrating the acceleration data.

(1) $$\begin{array}{cc} \; & l_{n}=K_{n}*\sqrt[{4}]{a_{max,n}-a_{min,n}} \end{array}$$

#### 3.1.3. Heading Estimation

The heading was estimated by fusing the gyroscope and magnetometer heading. The fused heading is a weighted average of the current magnetometer heading, current gyroscope heading and previous fused heading. The weight coefficients are adaptive, based on magnetometer stability and the correlation of the magnetometer and gyroscope. For more details, see [17].

### 3.2. WiFi RSS Aided Localisation

As illustrated in Figure 1, the predicted RSS from radio maps and the calibrated RSS from the smartphone were used as the input in the BPF and floor number detection algorithms. Both algorithms calculate a new output for each step detection. Therefore, all RSS measurements since the last step are buffered until the next step and then used as one RSS vector. RSS values from the same AP were averaged because they were measured at roughly the same position. The radio maps were constructed with the WiCA Heuristic Indoor Propagation Prediction (WHIPP) tool [61]. The user can upload an image of the floor plan, draw the walls over the image and enter the scale, the building materials of each wall, as well as the location and other parameters of the WiFi APs. The tool then estimates the path loss for each AP on a grid of coordinates based on the location of the APs and the locations and materials of each wall. The path loss model incorporates the distance, wall attenuations and diffraction around corners. As such, it also provides good path loss estimations in Non-Line-Of-Sight conditions (NLOS). The model was extensively explained in [62]. The RSS values are finally predicted by subtracting the path loss PL (in dB) from the transmitted power *P*. *P* also accounts for the antenna gains of the AP and smartphone. These are often unknown, which translates to a bias of several dB in the predicted RSS values compared to the real RSS values. The RSS bias $RSS_{b}^{AP}$ was compensated with self-calibration per AP, as proposed in [63]. This method estimates the bias by mapping the Cumulative Distribution Functions (CDFs) of the measured RSS values to the CDFs of the estimated RSS values. For optimal calibration, a random walk through the building was made during the offline phase while recording the RSS, and then, the bias for each AP was calculated. In the online phase, each measured RSS value was calibrated by subtracting the bias for the corresponding AP.

### 3.3. Floor Number Detection

**Figure 2:**
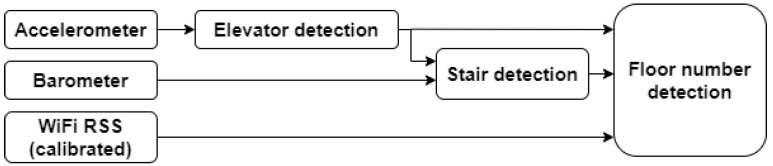
High-level flowgraph of the floor number detection algorithm. This algorithm consists of three phases. First, stairs and elevator usage are detected, and the height change is estimated. Then, RSS measurements are matched with the model-based radio maps, and finally, the output of the first 2 phases is combined to estimate the floor number.

Figure 2 shows a high-level flowgraph of the three-phase algorithm. It consisted of a combination of floor number detection with WiFi RSS MBF and floor transition detection using the accelerometer and barometer. In contrast to many comparable systems [23,25,28,29,55,56], our system detected both stairs and elevator usage, and these detections served a dual purpose: to aid in determining the sequence of visited floors (Section 3.3) and to improve 2D localisation (Section 3.4.3).

In the first phase, the accelerometer data were used to detect elevator usage. In the second phase, barometer data were used to detect stairs usage, while the (absence of) elevator detections was used to ignore noisy barometer data and prevent confusion between elevator and stairs detections. In the third and final phase, the output of the previous phases was fused with RSS fingerprinting by a Viterbi-based algorithm to detect the correct floor number.

RSS fingerprinting provides absolute floor number detection, but is prone to errors, and the number of APs per floor is limited (Section 3.5). Fusion with floor transition detection allowed the system to ignore false floor changes and also allowed detecting real floor changes earlier.

#### 3.3.1. Elevator Detection with the Accelerometer

**Figure 3:**
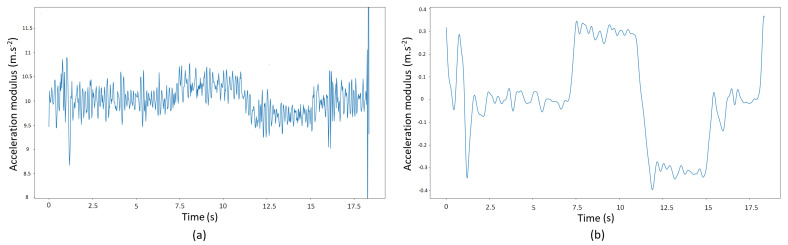
Acceleration modulus while the pedestrian is taking an elevator. Before (**a**) and after (**b**) filtering with the Gaussian low-pass filter and removal of the gravity component.

An algorithm for recognizing a moving elevator in accelerometer data was proposed, based on the elevator acceleration sensing principle of [64]. Our algorithm added a Low-Pass (LP) filter to the acceleration modulus to remove high-frequency noise, thus reducing the false negative rate. The acceleration modulus while the elevator is rising is shown before (Figure 3a) and after filtering (Figure 3b). These accelerations were measured with a Samsung Galaxy S4 Mini. To reduce the false positive rate, the algorithm *must* detect a hill followed by a valley to detect a rising elevator or vice versa. A maximum time offset between a candidate hill and valley was set by measuring (or estimating) the time the elevator needs to move from the lowest to the highest floor. In the case of an elevator detection, the first step after the detected elevator interval was labelled as ELEVATOR (Algorithm 1). In [64], the height change was estimated by double integration of the accelerations. However, integration errors can cause large deviations from the real height change. We estimated the maximum speed $v_{max}$ and acceleration/deceleration time interval of the elevator $T_{acce}^{elev}$ by averaging these two parameters from detected elevator transitions in the training data. The amount of floors changed *n* was then estimated using Equation (2), with $T_{total}^{elev}$ the time interval of the whole elevator transition and $h_{floor}$ the estimated floor height. This method provided better height estimation, at the cost of a limited amount of training data needed. For more details on elevator detection, see [64].
**Algorithm 1** Floor transition detection.
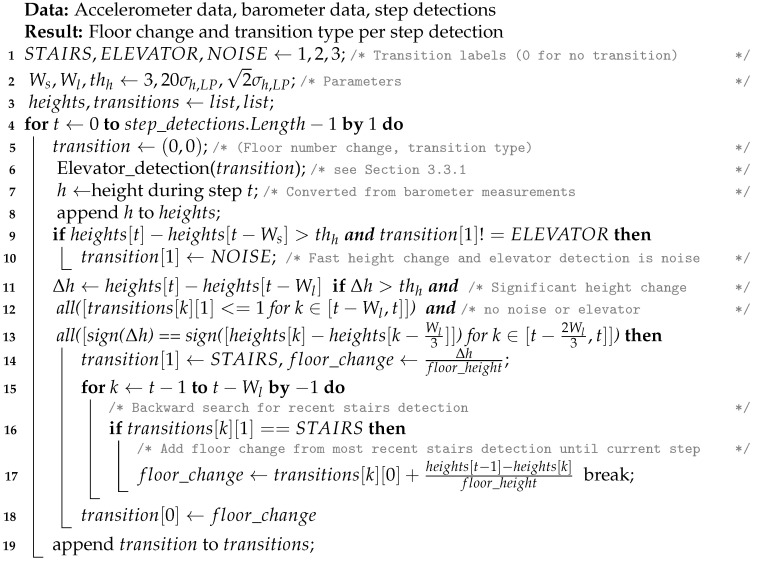

(2) $$n=\frac{v_{max}*\left(T_{total}^{elev}-T_{acce}^{elev}\right)}{h_{floor}}$$

#### 3.3.2. Stairs Detection with Barometer

In the stairs detection algorithm, the pressure was converted to a height change [52], but no initial height was assumed (i.e., the initial height was 0 m). Stairs transitions were detected by searching for a significant height change within a long window of $W_{l}$ step detections and a short window of $W_{s}<W_{l}$ step detections. Slow height changes (long window) indicated stairs usage, while fast height changes (short window) indicated either elevator usage or noise. This noise can be caused by opening/closing doors or windows [30]. The last step of a short window was labelled as NOISE (Algorithm 1) when a significant height change was detected within that window. All steps in a long window were labelled as STAIRS when a significant height change was detected within that window and none of the steps were labelled as ELEVATOR or as NOISE. This prevented the algorithm from detecting an elevator and stairs transition at the same time and also prevented false stairs or elevator detections due to large sudden pressure (and thus height) changes. We also noticed that fluctuations could last several seconds, especially in an older device. Therefore, we imposed an additional condition on the stairs detection: the sign of the height change must be constant over a smaller window, which is slid over the (long) stairs detection window. This means that large fluctuations within the long detection window would not trigger a stairs detection.

If the stairs transition was initially detected, the height difference over the whole window was estimated. If one or more STAIRS labels existed within the window, the same stairs transition was still happening; thus, the height change until the most recent detection was added to the height difference between that detection and the current detection.

The pseudocode of the described algorithm is provided in Algorithm 1. It uses three parameters: $W_{l}$, $W_{s}$ and the height change threshold $h_{th}$. The barometer of an older smartphone (Samsung Galaxy S5) produces significantly more noise than the barometer of a newer smartphone (Samsung Galaxy S7). Therefore, $h_{th}$ was adapted to the device. The length of the stairs detection window was also adapted to the device, since there must also be a *real* height change to be able to detect it. Assuming the barometer sensor noise is Gaussian and the height measurements (converted from pressure measurements) have a standard deviation $\sigma_{h}$, the estimated height difference by subtracting two height measurements was also Gaussian: $N(\Delta h,\sigma_{\Delta h}^{2})$ with Δh the real height difference between the two measurements and $\sigma_{\Delta h}=\sqrt{2}\sigma_{baro}$. We imposed $\left|\Delta h\right|=3\sigma_{\Delta h}$, and assuming a stairs rise of 0.15 m [65], the stairs detection window should be at least $20*\sigma_{\Delta h}$ steps long. A longer window means it will take longer before a stairs transition can be detected. An LP filter was used on the height data, and the new standard deviation $\sigma_{\Delta h,LP}<\sigma_{\Delta h}$ reduced the window length. However, the LP filter itself also caused a delay [53]. Therefore, we recursively calculated the optimal cutoff frequency for the LP filter for each device. The chosen value for the stairs detection threshold made false positives more likely than false negatives, given the expected value for the height change ($3\sigma_{\Delta h,LP}$). However, a false positive stairs detection is not as bad as a false negative detection in this context, because the detected height change must be at least 50% of the height between two floors to trigger a floor number change.

#### 3.3.3. Viterbi-Based Floor Detection

Finally, we proposed a Viterbi-based algorithm to combine the detected stairs and elevator transitions with WiFi RSS fingerprinting, to enable accurate floor number detection. The pseudocode of the algorithm is shown in Algorithm 2, and the important variables and equations are explained below. Note that Algorithms 1 and 2 are explained separately for clarity, but actually form one integrated algorithm.
**Algorithm 2** Viterbi floor detection.
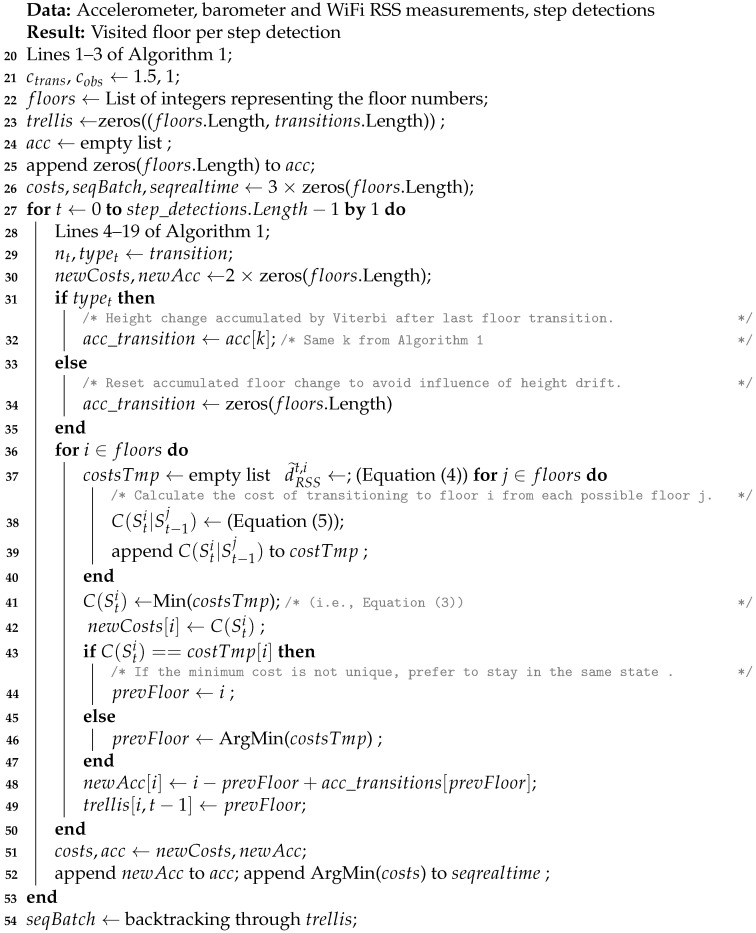

The original Viterbi algorithm [66] uses a transition and emission probability matrix to calculate the probability for current state $S_{t}^{j}$ given the previous state $S_{t-1}^{i}$, where *t* denotes time and i,j are the indices of possible states. On the contrary, our algorithm uses a cost function (Equation (5)) to calculate the cost $C(S_{t}^{j}|S_{t-1}^{i})$ for state $S_{t}^{j}$ given previous state $S_{t-1}^{i}$, where $S_{t}^{i}$ is the *i*-th floor during the *t*-th detected step index. $C(S_{t}^{i})$ is the cost up until the current step, given that the pedestrian is at the *i*-th floor (Equation (3)). The initial cost for each floor was zero, i.e., no assumptions about the initial floor were made. $n_{t}$ is the number of floor changes detected at step t. $type_{t}$ is the transition type at step *t*. $acc\_transition[S_{t-1}^{j}]$ is the floor change accumulated by the Viterbi algorithm during a stairs transition for the *j*-th floor up until the *previous* step. ${\tilde{d}}_{RSS}^{t,j}$ is the normalized distance between the measured RSS vector and the *j*-th floor and is calculated with Equation (4), where $d_{RSS}^{t,j}$ is the smallest Euclidean distance between the measured RSS vector at time *t* and the fingerprints of floor *j*. If the measured RSS vector does not contain a value for a certain AP, then a default value of −100 dBm is inserted for that AP. If there are no RSS measurements available at step *t*, then ${\tilde{d}}_{RSS}^{t,j}$ is set to zero for all floors. The transition cost $c_{trans}$ and observation cost $c_{obs}$ are coefficients that depend on the accuracy of the transition detection and fingerprint matching, respectively. A separate KD-tree was used for every floor radio map to quickly find the fingerprint with smallest Euclidean distance. Finally, the sequence with the smallest cost was chosen as the most likely sequence for batch processing. For real-time processing, the floor with the smallest cost was chosen at each step.

(3) $$\begin{array}{cc} \; & C\left(S_{t}^{i}\right)=\left\{\begin{array}{cc} Min\left[C\left(S_{t}^{i}|S_{t-1}^{j}\right)\right]\;\forall \;j\in \left[0,n-1\right], & t>0\\ c_{obs}.{\tilde{d}}_{RSS}^{t,i}, & t=0 \end{array}\right. \end{array}$$

(4) $$\begin{array}{cc} \; & {\tilde{d}}_{RSS}^{t,i}=\frac{d_{RSS}^{t,i}}{\sum_{k=0}^{n-1}d_{RSS}^{t,k}} \end{array}$$

(5) $$\begin{array}{cc} \; & C\left(S_{t}^{i}|S_{t-1}^{j}\right)=C\left(S_{t-1}^{j}\right)+c_{trans}.\left|i-j+acc\_transition\left[S_{t-1}^{j}\right]-n_{t}\right|+c_{obs}.{\tilde{d}}_{RSS}^{t,i} \end{array}$$

### 3.4. Backtracking Particle Filter with Clustering

**Figure 4:**
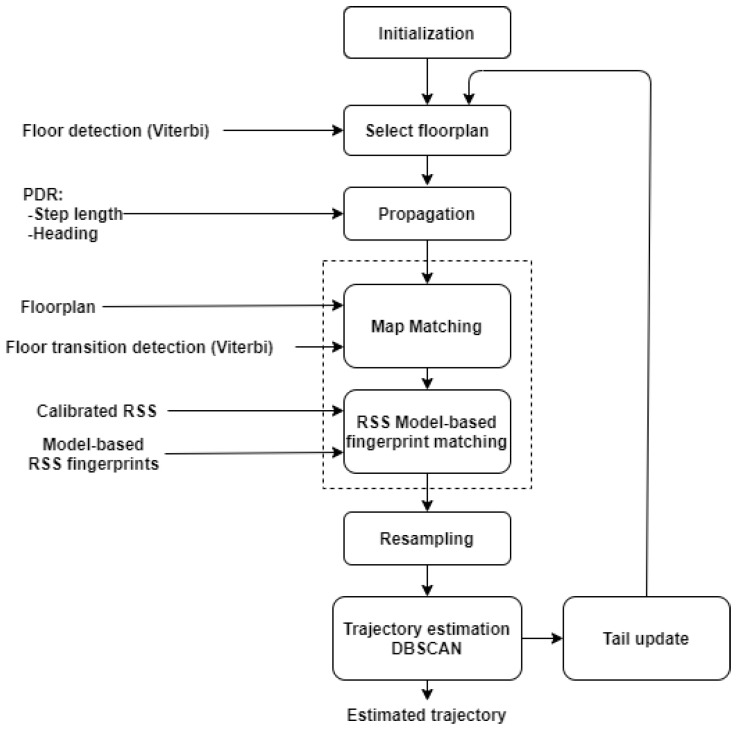
High-level flowgraph of the particle filter algorithm.

Figure 4 shows a high-level flowgraph of the BPF algorithm. The input for this algorithm is the output of all other parts of the system, i.e., PDR, floor plans, WiFi RSS fingerprinting, floor number detection and floor transition detection. Specific design choices are described here. For more details on BPF and PF in general, see [32,67].

#### 3.4.1. Initialization

First, the floor level was estimated using measured RSS values (Section 3.3), and the corresponding floor plan was loaded. A set of $N_{0}$ particles was uniformly distributed over the floor plan, each of which had the following attributes: $\left[x,y,b_{l},b_{h},W,parent\right]$. (x,y) is the 2D location of the particle. Step length bias $b_{l}$ and heading bias $b_{h}$ were randomly chosen. *W* is the particle weight and was set to $\frac{1}{N}$. parent points to the particle from which the particle originated and allowed tracing back its lineage recursively. parent was initially void.

#### 3.4.2. Propagation

First, the BPF checks if the floor number has changed and loads the new floor plan if necessary. The particles from initialization or from the previous cycle (Figure 4) are then deeply copied. These copies are the new particle generation, and parent now points to the original particle.

Propagation of the *i*-th particle was performed by calculating the position of each new particle based on the parent location, inherited biases and the current PDR output. Artificial Gaussian noise $N(0,\sigma_{h})$ was added to the inherited heading bias $b_{h}^{i}$ to enable heading drift compensation. Although $N(0,\sigma_{l})$ was added to $b_{l}^{i}$ to account for noise, $b_{l}^{i}$ was inherited without the noise, because a systematic step length error was assumed. The latter occurred due to badly tuned coefficients in the step length model. More details on the propagation model can be found in [19,20].

#### 3.4.3. Update

As depicted in Figure 4, the floor plan, WiFi RSS measurements (Section 3.2), elevator detections (Section 3.3.1) and stairs detections (Section 3.3.2) were used to reweigh the particles. If the trajectory between a particle and its parent intersected a wall segment, the particle was removed. This also removed the reference to its predecessors. If all references to a particle of a previous step were removed, that particle was lost. This way, the BPF was able to smooth the trajectory on the fly. The (B)PF algorithm is easily expanded by fusing additional information.

If a calibrated RSS vector was available, a new weight $W_{i,new}$ was assigned to each particle *i* by comparing the “measured” vector with the database RSS vector at the position of the particle with Equation (6), where $W_{i,old}$ is the particle’s old weight, $\sigma_{rss}$ is the standard deviation of the RSS noise and d is the Euclidean distance between the vectors. $\sigma_{rss}$ depends on the quality of the PL estimation and RSS stability and was empirically determined. Furthermore, the database vector was adapted to the measured vector, depending on the APs from which the signals were received.

Furthermore, $W_{i,new}$ was reduced when particle *i* was not on a staircase while the step was labelled as STAIRS. The same applied for the ELEVATOR label.

(6) $$\begin{array}{cc} \; & W_{i,new}=W_{i,old}\cdot e^{\left(\frac{-d^{2}}{2\sigma_{rss}}\right)} \end{array}$$

#### 3.4.4. Resampling

If the amount of particles dropped below $N<<N_{0}$, the weights of the updated particles were normalized, and a new set of *N* particles with equal weights was created by sampling from the updated particle set. The high amount of initial particles $N_{0}$ was only needed to have many particles spread over the floor plan. When the filter converged, a smaller particle set sufficed and was more efficient.

#### 3.4.5. Trajectory Estimation with DBSCAN

As mentioned before, multiple physically valid solutions are possible depending on the travelled trajectory, the geometry of the building, the amount of APs available for fingerprinting, etc. This leads to multimodal state distributions, i.e., the particles are gathered in different clusters. This happens especially when no constraints on initial position and/or heading are applied [26], as was the case in our system. Simply using the centroid of the particles causes large errors. Therefore, DBSCAN [33] was proposed to recognize the largest cluster of a particle set. The motivation for using DBSCAN was that the amount of clusters can change and must be detected automatically. Since the walls were used to remove impossible particles, the clusters can be arbitrarily shaped. DBSCAN can handle both situations and can also detect outliers by excluding them from any cluster. All obtained clusters were refined by removing the particles that had a wall between them and the cluster centroid. The removed particles were annexed to a new cluster. The position was finally estimated by calculating the weighted centroid of the largest cluster if its weight was at least 75% larger than the second “heaviest” cluster. If only one cluster was found, it had to contain at least a third of all particles. In all other cases, the position was estimated as the weighted centroid of all particles. While these parameters were not very sensitive, choosing much lower values increased the chance of choosing the wrong cluster because the correct cluster was not always the largest. Especially if the PDR output was noisy, the correct cluster would often remain small until particles of other clusters started to disappear because of wall collisions.

If a cluster was found that satisfied the weight conditions, the weights of its particles were increased by 5%. Choosing a higher fraction can lead to sample impoverishment in case the wrong cluster is chosen. To increase particle diversity when multiple similar clusters were found, the particle weights were increased by a factor inversely proportional to the cluster weight. This would increase the chance that particles of small clusters would be resampled and that a small, but correct cluster would “survive” long enough until other larger clusters bumped into the walls. The particle weight increase was again limited to a maximum of 5% to prevent the overinflation of the weights.

**Figure 5:**
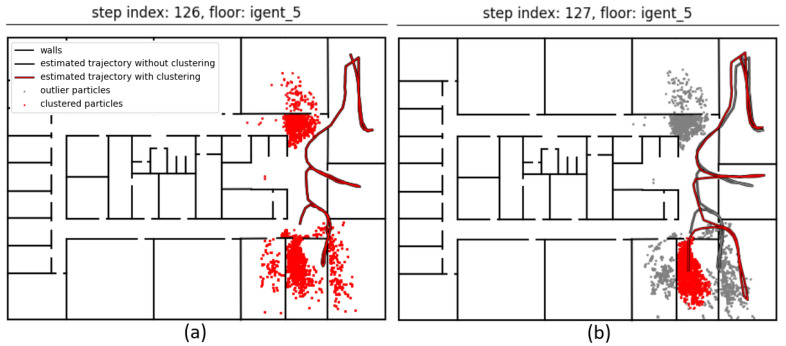
No significantly heavy cluster was found (**a**); thus the new position was calculated by averaging all particles. One step later (**b**), the correct cluster became heavier and was recognized by the clustering algorithm. The new position was calculated by averaging only the clustered (red) particles.

The BPF updated the previous positions by recursively calculating the centroid of the predecessors of only the cluster’s particles. This allowed the BPF to recover when the wrong cluster was chosen and was removed during subsequent map matching steps. In that case, another cluster would be chosen, and the previous positions could be corrected by backtracking. This is illustrated in Figure 5. Multiple clusters were found (Figure 5a), but none of them were significantly heavier than all other clusters. No particular cluster was selected; thus, the position (and the entire trajectory) was estimated using all particles. One step later (Figure 5b), the weight of the correct cluster increased and now exceeded the weight threshold. Only the clustered (red) particles were now used for calculating the new position and backtracking the previous states, resulting in the red trajectory. The other (grey) particles were considered to be outliers. The grey trajectory was estimated by backtracking with all particles. Note that the outlier particles were not deleted. Instead, all particles that were chosen during the resampling step were used in the next filtering cycle to prevent sample impoverishment.

#### 3.4.6. Tail Update

The *tail* is the sequence of particle generations that are still recursively linked to the present particle generation. If the tail length has reached the given limit, the oldest particle generation is removed. This means the currently oldest position cannot be updated with backtracking any longer.

A short tail length requires less CPU time and memory, while a larger tail length provides higher accuracy [32].

### 3.5. Evaluation Configurations

#### 3.5.1. Environment

**Figure 6:**
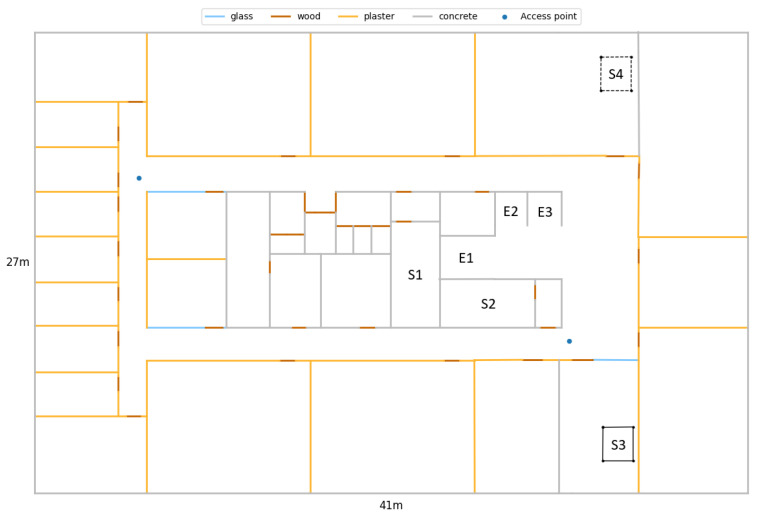
Floor plan of the fifth floor of the office building. E1–3 are elevators. S1–2 are closed concrete staircases that connect to all floors. S3 is an open metal staircase that connects the kitchens of the fifth and sixth floors. An identical staircase connects Floors 7 and 8. S4 is shown in dashed lines because it actually connects Floors 9 and 10. Two access points are located in the corridors. All other floors also have two access points in the corridors.

**Figure 7:**
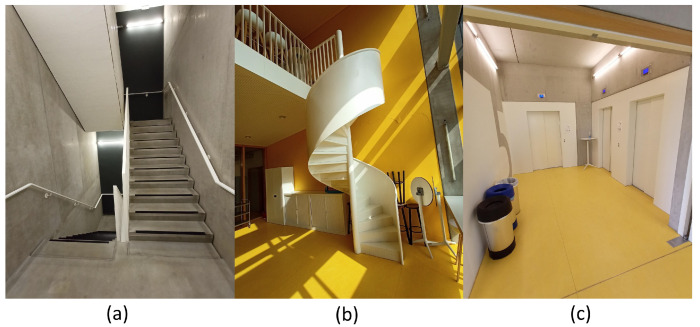
Pictures of one of the staircases in separate stairwells (**a**), the open staircase in the kitchen (**b**) and elevators (**c**).

The considered environment consisted of seven 41 m × 27 m (1107 m${}^{2}$) floors in an office building. Figure 6 shows a floor plan of the 5th floor of this building, where most of the measurements were performed. The office floor has three elevators (Es), four staircases (Ss) and two WiFi APs. Figure 7a shows one of the two centre staircases. These are located in separated stairwells and connect to all other floors of the twelve-storey building. Figure 7b shows one of the metal open spiral staircases, each of which connect the kitchens of two floors. S4 is indicated by dashed lines because the staircase is only present at Floors 9–12, while S3 is present at Floors 3–8. Figure 7c shows the three elevators. The APs are indicated with blue dots. Note that the centre of the floor consists of thick concrete walls, where the smartphones often cannot detect the APs’ signals. The floor height is 3.5 m. Plots of the other floors were omitted because they are almost identical. For example, each floor has two APs, but sometimes in a different corner of the hallways. The path loss model of Section 3.2 incorporated all elements of Figure 6, except for the open staircase (S3), when predicting the RSS fingerprints for the fifth floor.

#### 3.5.2. Validation Approach

The smartphones used were a Samsung Galaxy S5 (2014) and a Samsung Galaxy S7 (2016). Both smartphones have a 9-DOF IMU, barometer and WiFi chipset. The data were logged with the GetSensorDataApp [68]. The smartphone was placed on a stable surface for a few seconds at the start of each trajectory, then rotated around two perpendicular axes. This was performed to calibrate the gyroscope and magnetometer, respectively. The user held the smartphone in the hand without rotating it relative to the body. The interface of this app has a button for marking the time when the user passes a known position. The known positions were marked on the floor using tape and often lied in the middle of a hallway in front of a door frame for easy and correct annotation of the coordinates. The user pressed the button while stepping on the tape. For a trajectory with *n* known positions, *n* timestamps would be marked in the data. For each of these timestamps, the estimated position of the detected step closest in time was chosen. No interpolation between two estimated positions was performed. The errors of the localisation algorithm were determined by the Euclidean distance between the known positions and the selected estimated positions.

For multifloor trajectories, known positions were included in the elevators and at the entrance of the staircases to correctly evaluate the floor (transition) detection algorithm. The floor transition detection accuracy was determined by comparing the predicted labels for each step to the true labels. The possible labels were “Walking” (i.e., no transition detected) “Stairs Up”, “Stairs Down”, “Elevator Up” and “Elevator Down”. “Up” and “Down” denote positive and negative height change. This distinction is often made in the literature.

The floor detection errors were calculated as the absolute difference between the true and estimated floor for each step detection, but only when the true activity was Walking. Detected transition intervals during real walking did not cause ambiguity, since the floor detection algorithm would estimate the most likely floor number regardless of the activity.

#### 3.5.3. Algorithm Configurations

One 80 m long trajectory was travelled by one person in the same environment using both phones, to select the parameters for the BPF algorithm and to determine the barometer noise parameter for each device. To calibrate the elevator detection algorithm, one person used the elevator several times, rising/descending a different amount of floors each time. The parameters and their corresponding values are listed in Table 1. Parameters from other algorithms, such as the PDR algorithm, RSS prediction and elevator detection, were taken from referenced papers [17,41,52,62,64].

The accuracy of the BPF with DBSCAN clustering was compared to the default BPF, while the Viterbi-based floor detection algorithm was compared to the conventional approach: pure RSS matching using the Euclidean distance metric. Furthermore, to assess the contribution of fusing the RSS with transition detection, the algorithm was also tested without incorporating the transition detections. Lastly, both the BPF and floor detection algorithms could correct previous estimations based on new information and were therefore evaluated in real-time and batch mode.

#### 3.5.4. Trajectories

**Figure 8:**
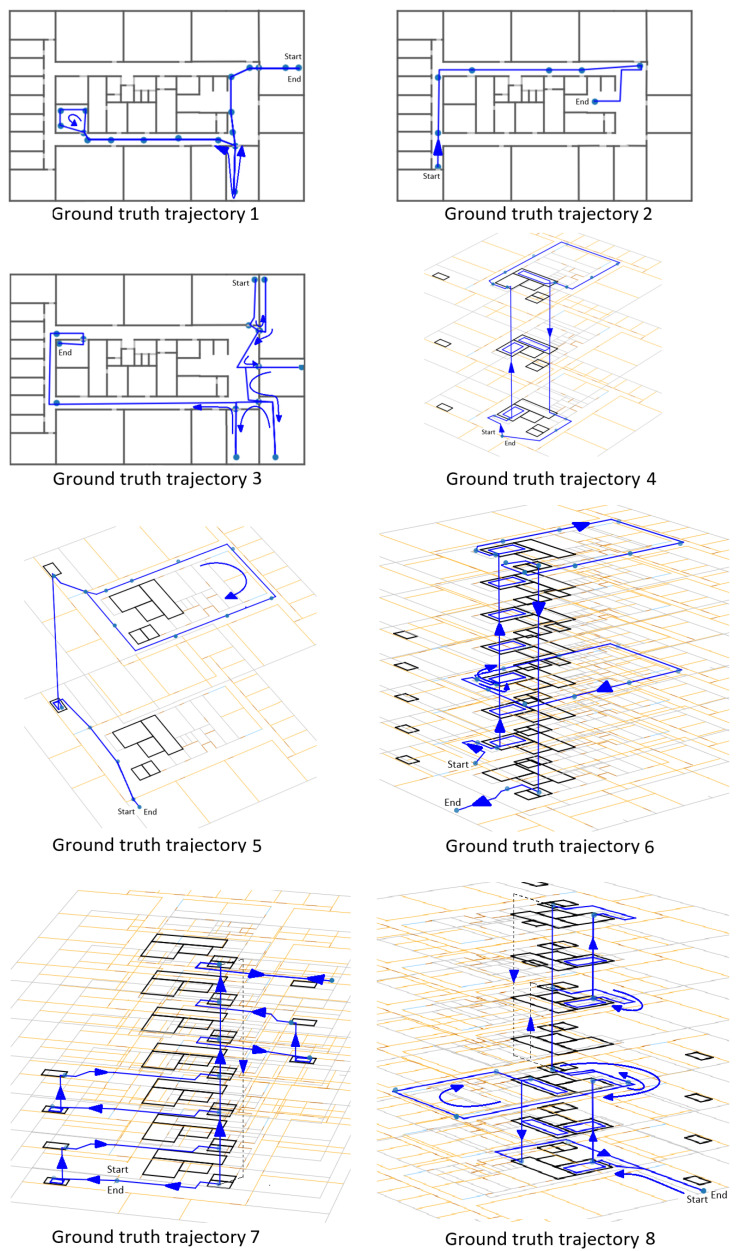
Evaluation trajectories: dots are the known positions used for evaluation. The first three trajectories are on the fifth floor only. The other trajectories are multifloor trajectories. The total length of all trajectories is 1.2 km.

Eight trajectories were travelled for the evaluation. Figure 8 shows the evaluation points of each trajectory. These trajectories had a combined length of 1230 m and consisted of a total of 160 known points and 19 floor transition events. We recorded a total of almost two hours (116 min) of data for the evaluation. The first trajectory was performed once by four persons, and all other trajectories were performed four times by one person, which resulted in a total travelled distance of 4.9 km and 76 floor transition events. The first three trajectories were entirely on the fifth floor, while the others were multifloor trajectories. In total, there were 12.6 min spent using the elevator and 23 min spent climbing the stairs. The remaining 81 min were spent roaming through the building, mostly on the fifth floor. More information for each trajectory is provided in Table 2.

## 4. Results

### 4.1. Floor Detection

Table 3 and Table 4 show the confusion matrices of the classification results for batch and real-time floor transition detection. In real-time mode, the most probable floor at a given time was estimated using only information from the past. In batch mode, the most probable floor was estimated by backtracking through the Trellis diagram (i.e., always using information of the whole trajectory). The global detection accuracy of these intervals was 90.9% (Table 3) for batch and 84.6% (Table 4) for real-time mode. These results were biased, since most of the evaluation data consisted of regular walking, and always choosing Walking would result in 69.5% accuracy. The unbiased global accuracies were 92.6% (batch) and 81.6% (real-time). In real-time mode, most errors were false negative stairs detections at the start of a stairs transition, because of the long detection windows where past measurements were used to detect the transitions. These steps were thus labelled as regular walking. This was a design choice, because the long detection windows allowed us to filter out noisy measurements. Indeed, the amount of false negative stairs detections was drastically reduced in batch mode, because we knew at the start of a stairs transition that the past steps in the detection window happened on a staircase as well. Of the remaining stairs detection errors, all false negatives and most of the false positives happened at the start and end of each real stairs transition as well, because of the delay introduced by the LP filter on the barometer data and the long detection window (Section 3.3.2). These errors had no impact on the floor number detection or localisation algorithms. The false negative errors at the start of stairs transitions in real-time mode had a minor impact on localisation: the BPF would converge slower if the state distribution was multimodal during those situations, since stairs detection could not be used to reduce the weights of the wrong particles (Section 3.4.3). The last kind of stairs transition errors could have an impact on the floor number detection accuracy: false positive stairs transitions that were not happening right before or after a real stairs transition. This happened mostly while using the Samsung Galaxy S5, which produced significantly more noise. As mentioned earlier, the detected height change must be at least 50% of the height between two floors to trigger a floor number change. Therefore, most of these false positive detections did not cause a floor number change. On the few occasions where a false detection did trigger a floor number change, the Viterbi algorithm added an extra transition back to the correct floor in a matter of seconds, because the RSS measurements favoured the correct floor. Although most false stairs detections did not trigger a floor number change, they could still influence the reweighing of particles. However, the chance that particles were in a staircase during a false detection was very small, because the staircases were confined spaces and particles could easily bump into walls. In any case, the weight penalty for particles outside staircases was kept lower than the penalty for particles outside of elevators.

**Figure 9:**
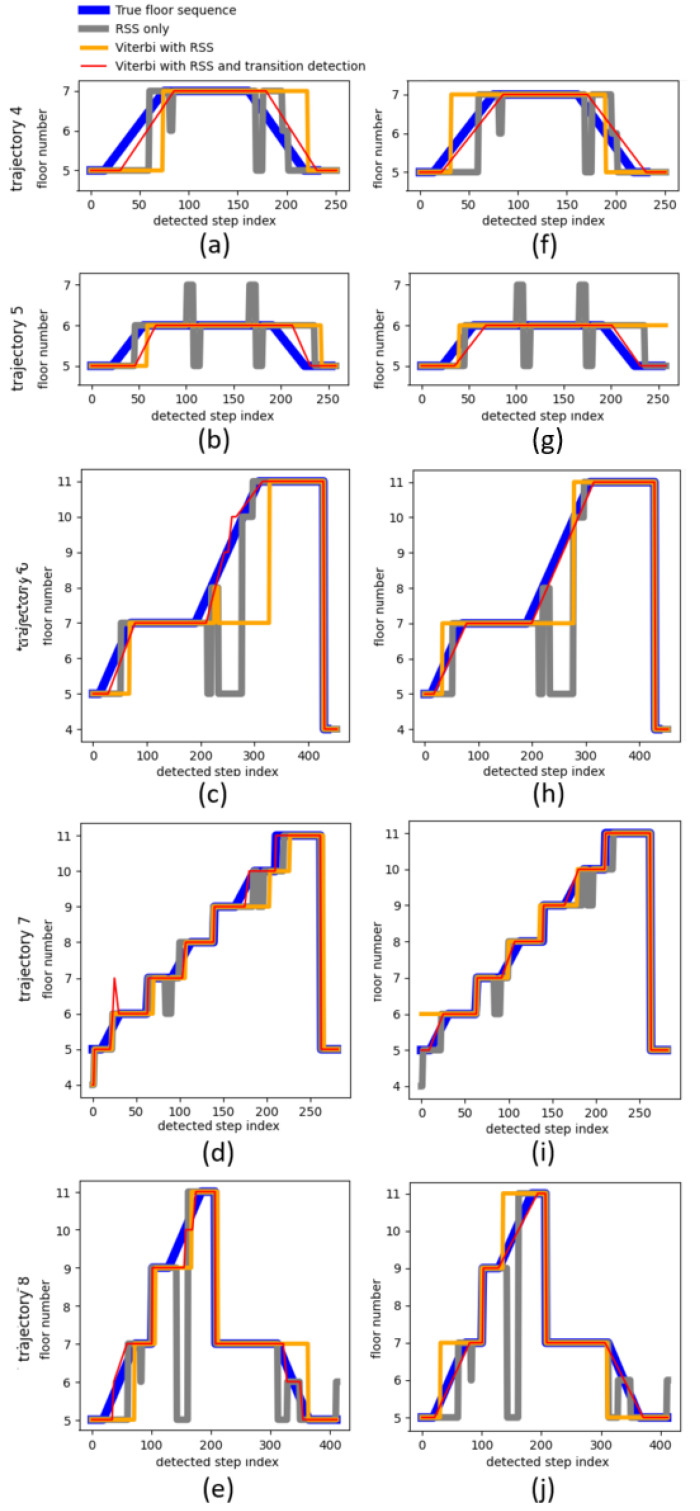
Plots of the estimated floor number as a function of the detected step index for variations of the floor number detection algorithm. (**a**–**e**) shows the real-time output for one iteration of each multifloor trajectory. (**f**–**j**) shows the batch output for the same iteration of the corresponding trajectories.

Table 5 lists a comparison of the variations of the floor number detection algorithm. As expected, the highest accuracy was achieved by fusing both transition detection and RSS measurements, with batch (99.7%) providing slightly better accuracy than real-time mode (94.1%). Surprisingly, the accuracy of the RSS only (91.6%) was higher than that of Viterbi with the RSS in real time (85.3%), while Viterbi with RSS in batch mode (94.1%) lied in the middle of the five configurations. The results of one iteration of each multifloor trajectory are visualized in Figure 9. Figure 9a–e shows the real-time output for one iteration of each multifloor trajectory. Figure 9f–j shows the batch output for the same iterations of the corresponding trajectories. These plots confirmed the explained results. It is also visible that real-time Viterbi with the RSS was a smoothed version of the RSS only, but with a significant delay. This delay explained why it failed to improve the floor number detection accuracy compared to the RSS only. Furthermore, in Figure 9d, the estimated floor number was initially wrong. This could happen in the case of bad RSS measurements, since the floor number was initially estimated by the RSS matching only (Section 3.3.3). The estimated height change during the first stairs transition was also too large and caused an overshoot. However, the Viterbi algorithm successfully added two extra transitions to compensate for this.

With over 99% floor detection accuracy, our algorithm was comparable to other recent works [31,53]. However, both required the initial floor to be known. The floor transition detection in [53] was designed for a specific type of staircase, where each floor transition consisted of “two staircases and a transition area between them”. Reference [31] achieved 100% accuracy, but a newer high-end smartphone (iPhone X) provided a clear advantage in detecting the correct floor number. Other systems (e.g., [23,24]) were difficult to compare because the floor detection accuracy was not separately evaluated.

### 4.2. Localisation

**Figure 10:**
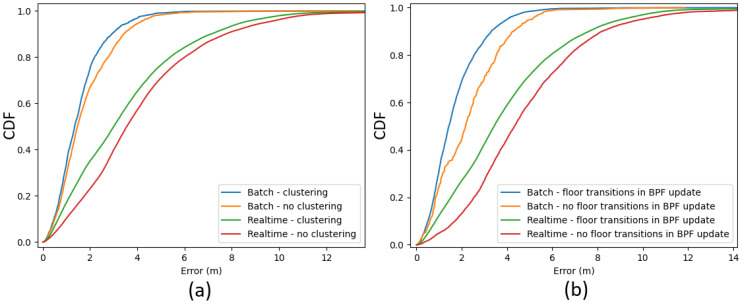
Error CDFs of the backtracking particle filter algorithm in real-time and batch mode. (**a**) Comparison between localisation with and without clustering, for all trajectories. Floor transition detections and the WiFi RSS were used as measurement updates in the BPF algorithm for each configuration. (**b**) Comparison between using floor transition detections as measurement updates in the BPF algorithm and not using the transition detections, for all multifloor trajectories. The proposed clustering algorithm was enabled, and the WiFi RSS was used as the measurement update.

Figure 10a shows error CDFs comparing the BPF with clustering to the BPF without clustering, both in real-time and batch mode. Each CDF was based on the errors of all (32) evaluation recordings, each processed ten times. All configurations used WiFi RSS and activity (stairs and elevator usage) detections as measurement updates. Some error statistics of these results are summarized in Table 6. The proposed clustering algorithm on top of the BPF reduced the median error of the overall recordings (each run 10 times) by 17% (real-time) and 13% (batch) compared to the conventional BPF. The 90th percentile error was reduced by 8% (real-time) and 15% (batch). The large performance difference between real-time and batch mode was partly due to the BPF being initialized with all particles uniformly distributed. The algorithm needed time to converge; thus, the real-time position error was always larger during the first 20–25 step detections.

**Figure 11:**
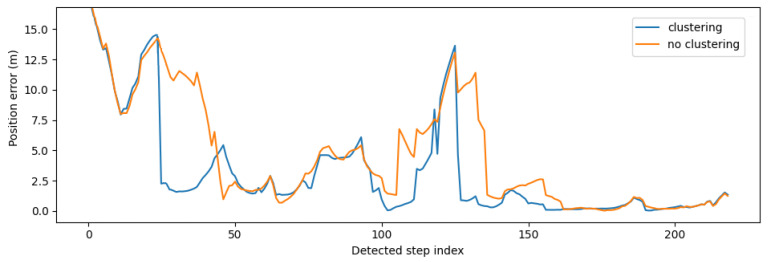
Real-time position error with (blue) and without (orange) the proposed clustering algorithm on top of the (backtracking) particle filter as a function of the detected step index, for Trajectory 6.

Figure 11 is an example of the real-time position error for one recording of Trajectory 6, comparing the proposed clustering algorithm on top of the BPF to the default BPF. Since there were only a few ground truth positions for each trajectory, the errors of this visualization were calculated by interpolating new positions for the detected steps. If *n* steps were detected between two ground truth positions (marked in the recording by pressing a button in the Android app), then *n* positions were interpolated on the blue lines in Figure 8 connecting the two ground truth positions. The proposed clustering algorithm produced the same or better results most of the time in this example (Figure 11). However, for some trajectories, the accuracy was not improved or even slightly reduced.

A different approach to solving multimodality was proposed in [23]. Similarly, their method was shown to improve accuracy in some situations, but reduced accuracy in other situations. However, no global improvement was achieved in [23], while our method achieved significant improvement. Note that, initially, the user had no knowledge on the starting position, apart from the rough estimation provided by RSS fingerprinting with very low AP density. After the user started walking, the IMU data were processed by the PDR algorithm, of which the output was further processed by the BPF. After some steps, many particles were removed due to wall intersections and RSS measurements. The remaining particles were resampled many times, thus creating clusters. Around 20–30 steps, a relatively large cluster was formed around the true position. The algorithm recognized that this cluster was larger than other clusters and calculated the position by averaging the particles of this cluster only. This resulted in a sudden reduction of the error for the clustering method, while the conventional method took longer to converge.

**Figure 12:**
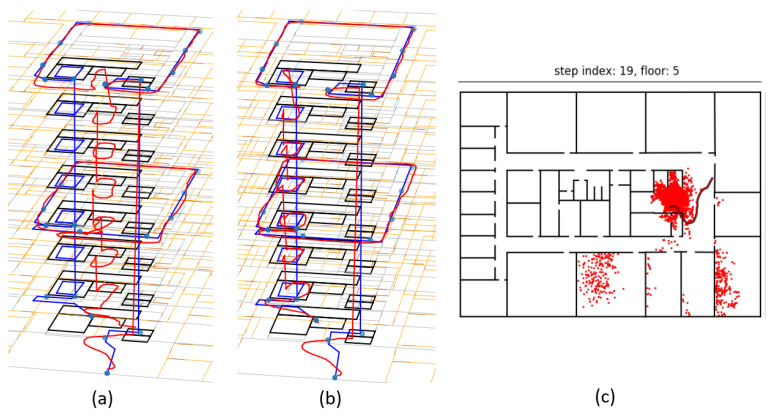
Plot of the estimated trajectory (red) and true trajectory (blue) by the backtracking particle filter in batch mode with (**a**) and without (**b**) using stairs detections as measurement updates. The distribution of particles when entering the staircase (**c**) is the same in both cases.

As mentioned in Section 3.4.3 and Section 3.3, floor transition detections were used as measurements updates by the BPF algorithm to improve 2D localisation. More specifically, the particles were reweighted by reducing the weights of the particles outside of any staircase or elevator in the case of a corresponding detection. Figure 10b shows error CDFs comparing the BPF using detected floor transitions as measurement updates with the BPF without using the floor transitions, again in real-time and batch mode. The CDFs were based on the errors of only the multifloor trajectory recordings, each processed ten times. The proposed clustering algorithm and WiFi RSS measurement updates were enabled for all four configurations. This means the blue curves in Figure 10a,b represent the localisation error CDF for identical configuration, but the former was based on all trajectories. The median error was reduced by 20% in real-time and 37% in batch mode. The advantage of reweighing the particles based on stairs detections is explained in Figure 12c: when the pedestrian entered the staircase, only a handful of particles entered the closed staircase, while most of the particles were gathered in front of the elevator. While the pedestrian walked up or down the stairs, many particles ran into the walls of the staircase. At the same time, the particles in the open area in front of the elevators did not run into walls as often. Without reweighing the particles when the stairs were detected, the particles inside the staircase would not survive. Figure 12a,b shows the batch results with and without reweighing the particles during stairs transition detections, respectively. The correct trajectory was successfully backtracked in (Figure 12a), while there was a position offset towards the elevators while the pedestrian was walking up the stairs in (Figure 12b).

## 5. Discussion

We presented an indoor smartphone-based localisation system for pedestrians, which consisted of a clustering algorithm based on DBSCAN on top of a backtracking particle filter and a new Viterbi-based floor number detection algorithm. The clustering algorithm attempted to solve multimodality, which is a known problem in the particle filtering context, while the floor number detection algorithm was developed to extend our system from 2D to multifloor localisation. The WiFi RSS measurements were fused with detected floor transition events. The RSS measurements were matched to a model-based radio map of each floor to provide absolute floor number estimation. These radio maps were made using the WHIPP tool [61,62]. The detected floor transitions provided accurate estimations of height changes or the lack thereof, allowing fast detection of floor transitions while being able to ignore false transitions caused by noisy RSS measurements. Our system was able to detect and estimate the height change during stairs and elevator usage. We implemented an existing elevator detection algorithm [64] and added a constraint to reduce the chance of false detections. Then, we developed a new adaptive stairs detection algorithm, which changed its parameters according to the noise produced by the barometer sensor. Our stairs detection algorithm also addressed the pressure drift problem and was able to ignore fast pressure changes, which could be caused by opening/closing doors or windows [30]. Unlike comparable algorithms ([51,56]), our floor number detection algorithm worked separately from the particle filter algorithm. This avoided conflicts when there were no particles at a staircase. However, it was possible in our localisation system that the floor number changed while the estimated position was outside of a staircase or elevator (Figure 12). In addition, the RSS radio maps were also used to speed up the convergence of the particle filter during initialisation.

Our system was evaluated by real measurements spanning seven 1107 m${}^{2}$ floors in an office environment (Section 3.5). Each floor had two APs, resulting in a sparse AP density compared to other systems in the literature. These APs were part of the wireless network of the office, so no APs were specifically installed for our training or evaluation measurements. Furthermore, the walls in the centre of the building (surrounding two staircases) were made of concrete, which often blocked the WiFi signal completely. We travelled eight different evaluation trajectories and repeated them each four times using two smartphone types, resulting in almost two hours of sensor data and a total travelled distance of 4.9 km. Five trajectories included floor transitions, each of which was repeated four times, resulting in a total of 76 floor transition events. Our system was evaluated in both real-time and batch mode, since the Viterbi and backtracking particle filter algorithms could improve past position estimations with new information.

The median position error of our localisation system was 3.0 m in real-time and 1.3 m in batch mode. The proposed clustering algorithm was shown to reduce the median error by 17% (real-time) and 13% (batch) compared to the same localisation algorithm without clustering. All stairs and elevator transitions were detected. False stairs transitions were detected several times, which resulted in an overall activity (i.e., walking, stairs up, stairs down, elevator up, elevator down) recognition accuracy of 84.6% (real-time) and 80.9% (batch). However, most false transition detections had no impact on the performance of the floor number detection algorithm, which was able to ignore these false detections. In the few cases where the algorithm wrongly changed the floor number, it switched back to the correct floor several seconds later. Sometimes, the height change during a real floor transition was estimated too low or high, which temporarily caused an error in the floor number estimation. These errors only lasted for a few seconds, until the algorithm added an extra transition towards the correct floor. The resulting accuracy of our floor number detection algorithm was 99.1% (real-time) and 99.7% (batch), while the accuracy of detecting the floor number using the RSS measurements only was 91.6%. Detecting floor transitions also allowed us to improve the 2D localisation by reweighing particles outside of staircases/elevators during detected transitions. This reduced the median error by 20% (real-time) and 36% (batch) for the multifloor trajectories.

Our algorithm was also practical: the RSS radio map was model based; thus, expensive measurement campaigns were not needed. We did not install additional hardware, e.g., a reference barometer [54], and only used pre-existing APs, making the tracked smartphone the only extra hardware needed. A small amount of training data per device was needed for calibration of the WiFi RSS-MBF and floor transition algorithms. However, this was easily performed by simply walking a random trajectory (50–100 m was enough for our environment) and taking the elevator a couple of times.

Two ways to improve this system were identified. First, the magnetometer was only calibrated at the start of each trajectory. However, disturbances from external magnetic objects depend on the user location. The work in [69] proposed a simple calibration method for compensating these disturbances while walking. This method could be used here to improve the PDR algorithm. Second, while our MBF path loss model was accurate in NLOS conditions by incorporating the floor plan, we did not compensate for human body influences. As shown in [70], the human body standing between the smartphone and an AP could easily increase the path loss by 10 dB. Orientation-aware fingerprinting would allow the BPF to rely more on the RSS and improve localisation accuracy, especially during the initial stage when the particles are still spread out.

To deploy this system in a new environment, some pre-existing APs and a detailed floor plan (including AP locations and building materials) must be available. The following steps need to be performed to set up the system:

- The radio maps were constructed using the WHIPP tool (Section 3.2);
- A random walk was performed on each floor to calibrate the radio maps (Section 3.2). The data from these walks were also used to calibrate the stairs detection algorithm (Section 3.3.2);
- The elevator was taken several times, and the floor change was annotated, to determine the parameters used to estimate the height change (Section 3.3.1);
- A short trajectory (80 m for our tests) of a known length was walked to tune the step length model (Section 3.1).

## 6. Conclusions

We designed an easily deployable multi-smartphone-based indoor localisation system for pedestrians. It required only a few WiFi APs, which are currently available in most buildings, and a smartphone. It also needed a limited and easily obtained amount of training data. We proposed a new floor detection algorithm, which detected stairs and elevator usage and fused these detections with WiFi RSS measurements to estimate the floor number. The proposed algorithm achieved 99.1% accuracy in real-time and 99.7% in batch mode. The evaluation dataset consisted of 116 minutes of recorded data, during which the actors changed floors 76 times and performed eight trajectories four times. A BPF estimated the travelled trajectory based on PDR, RSS measurements, floor transition detections and a floor plan. We proposed a clustering algorithm based on DBSCAN on top of the BPF to solve multimodality in the filter’s state distribution. While the problem was not entirely solved, the proposed algorithm reduced the median error by 17% in real-time and 13% in batch mode.

Our system was prone to another common problem in this research area: sample impoverishment. This problem manifests itself in particle filters when all particles are propagated towards the wrong position and are trapped because of hard constraints (i.e., walls). For some of our recorded trajectories, the filter failed many times before finding a trajectory. Furthermore, the smartphone was held firmly in front of the body during all experiments. Future work will consist of making our system more robust by handling multiple ways of carrying the smartphone and preventing sample impoverishment.

## Figures and Tables

**Table 1:** Important parameters for the localisation and floor detection algorithms. GS5 and GS7 are abbreviations for the Samsung Galaxy S5 and S7 smartphones, respectively.

Algorithm	Parameter	Value
BPF	$N_{0}$	20,000
BPF	*N*	1000
BPF	$\sigma_{l}$	0.15 m
BPF	$\sigma_{h}$	$3^{\circ }$
DBSCAN	ϵ	0.6
DBSCAN	minPts	0.05 N
WiFi RSS MBF	$\sigma_{rss}$	11 dB
Stairs detection	$W_{s}$	3
Stairs detection	$th_{\Delta h}$	0.8 m (GS5), 0.5 m (GS7)
Stairs detection	$W_{l}$	17 (GS5), 11 (GS7)
Floor detection	$c_{trans}$	4
Floor detection	$c_{obs}$	1

**Table 2:** Evaluation trajectory information. Each trajectory was repeated four times. “S” and “E” stand for stairs and elevator, respectively. * means the values are averaged.

Trajectory Details					Floor Details		
#	* Duration **(s)**	* Steps **Detected**	**Length**	**Evaluation** **Points**	**Floor Number** **Sequence**	**Transition** **Types**	* Transition **Time**
1	114	180	122	35	5	-	-
2	50	83	53	8	5	-	-
3	142	219	138	17	5	-	-
4	170	250	135	16	5, 7, 5	S2, S1	76
5	156	251	136	22	5, 6, 5	S3, S3	32
6	382	456	271	29	5, 7, 11, 4	S2, S2, E2	141
7	350	282	143	16	5, 6, 7, 8, 9, 10, 11, 5	S3, E3, S3, E3, S4, E3, E3	129
8	380	412	232	17	5, 7, 9, 11, 7, 5	S2, E3, S2, E3, S1	150

**Table 3:** Confusion matrix for batch floor transition detection.

		True Activity				
		**Walking**	**Stairs Up**	**Stairs Down**	**Elevator Up**	**Elevator Down**
Detected Activity	Walking	**89%**	5%	8%	3%	6%
	Stairs Up	6%	**95%**	0%	0%	0%
	Stairs Down	3%	0%	**92%**	0%	0%
	Elevator Up	1%	0%	0%	**97%**	0%
	Elevator Down	1%	0%	0%	0%	**94%**

**Table 4:** Confusion matrix for real-time floor transition detection.

		True Activity				
		**Walking**	**Stairs Up**	**Stairs Down**	**Elevator Up**	**Elevator Down**
Detected Activity	Walking	**89%**	35%	33%	3%	6%
	Stairs Up	6%	**65%**	0%	0%	0%
	Stairs Down	3%	0%	**67%**	0%	0%
	Elevator Up	1%	0%	0%	**97%**	0%
	Elevator Down	1%	0%	0%	0%	**94%**

**Table 5:** Floor detection errors for different configurations as distance in floor numbers to the true floor. The Viterbi-based algorithm incorporating both the RSS and floor transition detections achieved the highest accuracy.

		Difference between True and Detected Floor Number							
		**0**	**1**	**2**	**3**	**4**	**5**	**6**	**7**
Algorithm	RSS only (Euclidean distance metric)	**91.6%**	6.8%	0.2%	0%	1.0%	0.1%	0.3%	0.1%
	Viterbi: RSS (real-time)	**85.3%**	8.4%	3.5%	0%	2.1%	0%	0.5%	0.2%
	Viterbi: RSS (batch)	**94.1%**	3.8%	1.0%	0%	0.7%	0%	0.4%	0%
	Viterbi: RSS and floor transition detection (real-time)	**99.1%**	0.9%	0%	0%	0%	0%	0%	0%
	Viterbi: RSS and floor transition detection (batch)	**99.7%**	0.3%	0%	0%	0%	0%	0%	0%

**Table 6:** Error statistics of the backtracking particle filter algorithm with and without clustering, in real-time and batch mode.

	Mean (m)	P50 (m)	P75 (m)	P90 (m)
Batch—clustering	1.6	1.3	2.0	2.9
Batch—no clustering	1.8	1.5	2.5	3.4
Real-time—clustering	3.5	3.0	4.8	7.1
Real-time—no clustering	4.1	3.6	5.4	7.7

## Data Availability

Datasets are available on request.

## Sample Availability

Samples of the compounds are available from the authors.

## Abbreviations

The following abbreviations are used in this manuscript:

UWB	Ultra-Wideband
IMU	Inertial Measurement Unit
PDR	Pedestrian Dead Reckoning
PF	Particle Filter
KDE	Kernel Density Estimator
BPF	Backtracking Particle Filter
DBSCAN	Density-Based Spatial Clustering for Applications with Noise
RSS	Received Signal Strength
MBF	Model-Based Fingerprinting
AP	Access Point
AHRS	Attitude and Heading Reference System
WHIPP	WiCA Heuristic Indoor Propagation Prediction
NLOS	Non-Line-Of-Sight
CDF	Cumulative Distribution Function
LP	Low-Pass
